# Effect of single-port inflatable mediastinoscopy simultaneous laparoscopic-assisted radical esophagectomy on respiration and circulation

**DOI:** 10.1186/s13019-021-01671-z

**Published:** 2021-10-09

**Authors:** Haibo Ye, Xiaojin Wang, Xiaojian Li, Xiangfeng Gan, Hongcheng Zhong, Xiangwen Wu, Qingdong Cao

**Affiliations:** 1 Department of Thoracic Surgery, The Fifth Affiliated Hospital of Sun Yat-Sen University, 52 East Meihua Road, Xiangzhou District, Zhuhai, 519000 Guangdong China; 2 Department of Anesthesia, The Fifth Affiliated Hospital of Sun Yat-Sen University, 52 East Meihua Road, Xiangzhou District, Zhuhai, 519000 Guangdong China

**Keywords:** Esophagectomy, Inflatable mediastinoscopy, Circulation, Respiration

## Abstract

**Background and purpose:**

We previously developed a new surgical method, namely, single-port inflatable mediastinoscopy simultaneous laparoscopic-assisted radical esophagectomy. The purpose of this study was to evaluate the effect of carbon dioxide inflation on respiration and circulation using this approach.

**Methods:**

From April 2018 to October 2020, 105 patients underwent this novel surgical approach. The changes in respiratory and circulatory functions were reported when the mediastinal pressure and pneumoperitoneum pressure were 10 and 12 mmHg, respectively. Data on blood loss, operative time, and postoperative complications were also collected.

**Results:**

104 patients completed the operation successfully, except for 1 patient who was converted to thoracotomy because of intraoperative injury. During the operation, respectively, the heart rate, mean arterial pressure, central venous pressure, peak airway pressure, end-expiratory partial pressure of carbon dioxide and partial pressure of carbon dioxide increased in an admissibility range. The pH and oxygenation index decreased 1 h after inflation, but these values were all within a safe and acceptable range and restored to the baseline level after CO_2_ elimination. Postoperative complications included anastomotic fistula (8.6%), pleural effusion that needed to be treated (8.6%), chylothorax (0.9%), pneumonia (7.6%), arrhythmia (3.8%) and postoperative hoarseness (18.2%). There were no cases of perioperative death.

**Conclusions:**

When the inflation pressure in the mediastinum and abdomen was 10 mmHg and 12 mmHg, respectively, the inflation of carbon dioxide from single-port inflatable mediastinoscopy simultaneous laparoscopic-assisted radical esophagectomy did not cause serious changes in respiratory and circulatory function or increase perioperative complications.

## Introduction

Esophageal cancer (EC) is one of the most common malignant tumors in the world, ranking seventh in global morbidity and sixth in mortality [1]. The prognosis of EC is poor, and the 5-year survival rate is less than 25% [2]. Currently, the basic treatment strategy for EC is a combination of surgery, radiotherapy and chemotherapy, and surgical treatment is still the main and preferred treatment [2, 3]. Previous studies have shown that compared with open esophagectomy, minimally invasive esophagectomy (MIE) has the advantages of less postoperative pain, less intraoperative bleeding, shorter hospital stay and recovery time, and more extensive lymph node dissection. Thus, MIE has become the preferred surgical method [4–8].

The approaches of MIE are transthoracic and non-transthoracic (e.g., transhiatal esophagectomy). Transhiatal esophagectomy (THE) is considered to be less invasive and has fewer pulmonary complications because it avoids thoracotomy [9]. However, traditional THE has more stringent surgical indications because of its limited field of vision and insufficient mediastinal lymph node dissection, which is more suitable for cancers that indicate negative lymph nodes, and the oncology results are usually considered poor [10, 11]. To overcome this shortcoming, Fujiwara et al. developed a new surgical method, namely, a radical resection of thoracic EC by using inflatable mediastinoscopic lymph node dissection through cervical incisions and hand-assisted laparoscopic lymph node dissection of the upper mediastinum, and this method has been reported to be safe and feasible [12–14]. On this basis, our organization further improved the operation and carried out single-port inflatable mediastinoscopy simultaneous laparoscopic radical esophagectomy (SPIMSLE) [15].Our team improved this operation as following differences: first, our team is divided into mediastinal group and abdominal group to operate at the same time, and the operation time is significantly shortened; second, our abdominal group is operated completely under laparoscopy. rather than hand-assisted laparoscopic surgery, the trauma is less. Third, we have performed lymph node dissection of the right vagus nerve and the right recurrent laryngeal nerve through the left cervical incision [16].

In this procedure, we inflated the mediastinum and abdomen to provide a clear field of view for esophagectomy and mediastinal lymph node dissection. This operation, which is a relatively new surgical method, is not performed through the chest or one-lung ventilation and causes less damage to the lungs. Whether simultaneous inflation of the mediastinum and abdomen during surgery has a significant impact on the respiratory and circulatory systems has not been reported. The purpose of this study was to evaluate the perioperative safety of this procedure.

## Materials and methods

From April 2018 to October 2020, patients with esophageal malignant tumors were treated with SPIMSLE in the Fifth Affiliated Hospital of Sun Yat-sen University. The inclusion criteria were as follows: (1) pathologically confirmed malignant tumor and The preoperative T stages were T1b-T3; (2) functional tolerance of major organs and systems for radical surgery; (3) no other cancers; (4) no history of mediastinal surgery; The exclusion criteria were as follows: (1) cervical EC;(2) refusal of surgery.

This study was approved by the institutional review committee of our hospital. Written informed consent was obtained from each patient.

### Anesthesia

Preoperative medication included intramuscular injection of atropine 0.01 mg/kg. Anesthesia was induced by intravenous injection of midazolam 0.05 mg/Kg, propofol 2 mg/kg, muscle relaxant cis atracurium 0.15 ~ 0.25 mg/kg and sufentanil citrate 0.1–0.3ug/Kg. Insert 7/7.5-FR single lumen catheter (Sheridan endotracheal catheter, Sher-I-Bronch). Do not use bronchial blockers. Mechanical ventilation after intubation (tidal volume 6–8 ml / kg; inspiratory time / expiratory time ratio: 1 / 1.5). Adjust the respiratory rate and do not use positive end-expiratory pressure. Anesthesia was maintained by continuous intravenous infusion of remifentanil 0.25 g–1.0 g/kg/min, while inhaling air, oxygen and sevoflurane. After operation, the tube was extubated after the recovery of pharynx reflex, inhalation and ventilation. All intubation procedures are performed by experienced anesthesiologists.

### Surgical procedure

**Fig. 1:**
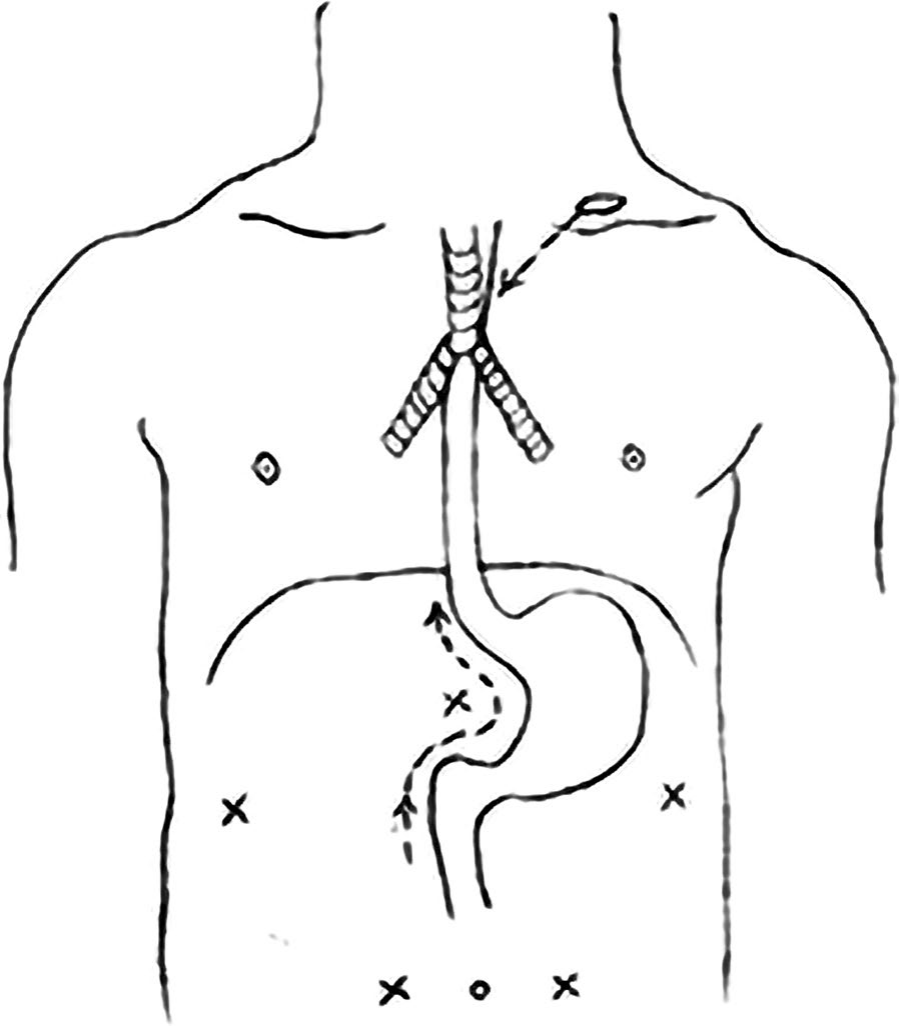
A brief view of the surgical procedure. Enter the upper mediastinum through the left supraclavicular incision in the neck. Five trocars are placed into the abdominal cavity through the five intersecting positions in the picture. The arrow is the approximate direction of the operation

The patients were ventilated with single-lumen endotracheal intubation under general anesthesia in the supine position; central venous pressure (CVP) was recorded by a central venous catheter in the right subclavian vein; and blood pressure was monitored by an indwelling arterial catheter in the right radial artery. As shown in Fig. 1, the operation was performed simultaneously in the mediastinum group and the abdominal cavity group. The mediastinum group entered the mediastinum through a cervical incision, and the abdominal group inserted a trocar into the abdominal cavity through five puncture points of the abdomen. The surgical incision, the location of the surgeon and the detailed surgical procedure are described in our recent report. [15, 17, 18].

The operation in the mediastinal group was performed as follows: first, access was gained through the cervical incision, and the cervical esophagus was freed, establishing a tunnel filled with carbon dioxide (CO_2_) gas to create artificial mediastinal emphysema and obtaining a pressure of 10 mmHg. After we methodically tried various pressure gradients, when the mediastinal pressure was 10 mmHg, the operative field was clear, and the operative space was wide. Second, we continued to dissociate the upper and middle thoracic esophagus and dissected the lymph nodes in the thoracic region.

The operation in the abdomen group was performed as follows: the artificial pneumoperitoneum pressure was 12 mmHg, which is the conventional laparoscopic operative pressure. The stomach was dissociated, and the lymph nodes in the abdominal region of the esophagus were dissected. Next, the lower thoracic esophagus was dissociated through the diaphragmatic esophageal hiatus, and the surrounding lymph nodes were cleared. Finally, it penetrated the superior mediastinum. The air source was turned off, and inflation was stopped.

The esophagus was incised at the neck, the Tubular stomach was created, and the neck was anastomosed with the proximal esophagus through the esophageal bed. The neck and abdominal incisions were sutured.

### Observation parameters

Heart rate (HR), invasive mean arterial pressure (MAP), central venous pressure (CVP), peak airway pressure (AP), end-expiratory CO_2_ partial pressure (ETCO_2_), inhaled oxygen concentration (FiO_2_) and tidal volume (TV) were continuously monitored and recorded during the operation. Arterial blood samples were collected for blood gas analysis at the following time points: 5 min before inflation of the mediastinum and abdomen (baseline); 1 h after inflation; 20 min after CO_2_ excretion; and 10 min after the operation. These data were collected from anesthetic records. At the same time, the operative duration, amount of bleeding, and A special event during the operation were recorded. Postoperative pathological data, postoperative complications, and postoperative hospital stay information were collected.

### Statistical analysis

Continuous data are expressed as the mean ± standard deviation (SD). The classified data are expressed as a percentage. During the operation, taking the pre-inflation data as the reference, the Welch test was used to analyze the numerical difference in each period after inflation. All statistical analyses were conducted using IBM SPSS version 25 (SPSS Statistics v25, IBM, Somers, NY, USA). When *P* < 0.05, the result was considered significant.

## Results

A total of 105 patients who underwent SPIMSLE from April 2018 to October 2020 were enrolled. The demographic and clinical characteristics of the 105 patients are summarized in Table 1. The average age was 62.6 ± 8.8 years (range 42–84 years), the percentage of vital capacity (VC) was 70.5–176.1% (101.68 ± 23.00%), the percentage of forced expiratory volume to predicted value within 1 s (FEV1%) was 67.9–163.3% (100.05 ± 20.80%), and the average cardiac ejection fraction was 69.35 ± 5.11. The tumor sites included the upper chest (n = 14), middle chest (n = 56) and lower chest (n = 35). The preoperative T stages were as follows: T1b, 36 patients; T2, 49patients; and T3, 20 patients. Comorbidities included COPD (n = 6), hypertension (n = 27) and diabetes (n = 9). Twenty-one of these patients received neoadjuvant chemotherapy.

**Table 1:** Patient demographics and clinical characteristics

Parameters	Number
Age	
Average (range)	62.6(42–84)
Gender	
Male	75
Female	30
Tumor location	
Upper thoracic segment	14
Middle thoracic	56
Lower thoracic	35
Biopsy pathology	
Squamous cell carcinoma	99
Adenocarcinoma	1
Small cell carcinoma	1
Sarcomatoid carcinoma	1
Neuroendocrine carcinoma	3
The preoperative T stages	
T1b	36
T2	49
T3	20
Concomitant disease	
COPD	6
Hypertension	27
Diabetes	9
Atrial fibrillation	2
Preoperative treatment	
Neoadjuvant chemotherapy	21
Neoadjuvant radiotherapy	0

### Intraoperative observation index

There were no deaths within 30 days postoperatively. A total of 104 patients underwent SPIMSLE, and 1 patient underwent thoracotomy because of tracheal membrane injury during mediastinoscopy. Pleural rupture occurred in 5 cases, and the airway pressure increased immediately after pleural rupture, up to 4 cmH_2_O. The effect of tension pneumothorax on the operation could be avoided by suspending the operation for several minutes, properly reducing tidal volume and enlarging the scope of rupture. The average operative time was 186.7 ± 43.9 min, and the average intraoperative blood loss was 113.4 ± 89.9 ml. The field of view was clear during the operation, and the average number of lymph nodes dissected was 22.5 ± 4.5.

During the operation, the hemodynamics and respiratory movement indexes of 104 cases were monitored when CO_2_ was inflated into the mediastinum and abdomen synchronously, as shown in Table 2. The data from 5 min before CO_2_ inflation were taken as the baseline, and there were significant differences in HR, MAP, CVP, Peak AP, ETCO_2_, PH, oxygenation index (OI, the ratio of inhaled oxygen concentration to the partial pressure of oxygen) and partial pressure of CO_2_ (PaCO_2_) after inflation.

**Table 2:** Changes in the respiratory and circulatory systems before and after inflation intraoperatively

		Inflatable stage					
	5 min before inflation (baseline)	5 min	10 min	20 min	1 h	20 min after gas shutdown	Postoperative 10 min
HR	69.1 ± 7.9	87.6 ± 15.5*	89.8 ± 16.7*	90.2 ± 16.5*	88.6 ± 14.3*	77.9 ± 12.8	73.0 ± 10.0
MAP	84.8 ± 6.4	103.4 ± 14.8*	104.3 ± 14.5*	97.9 ± 14.5*	98.2 ± 13.3*	90.6 ± 6.2	97.5 ± 10.6
CVP	6.3 ± 0.9	14.5 ± 1.5*	14.0 ± 1.9*	14.0 ± 1.6*	14.6 ± 1.7*	10.1 ± 1.6	9.0 ± 1.4
Peak AP	14.3 ± 1.6	21.7 ± 3.2*	23.1 ± 3.9*	23.0 ± 4.5*	23.4 ± 4.9*	15.5 ± 2.4	14.9 ± 2.2
ETCO_2_	33.4 ± 3.4	39.6 ± 3.3*	40.5 ± 5.8*	40.9 ± 5.9*	43.1 ± 5.6*	36.0 ± 3.4	34.6 ± 2.8
TV	403.2 ± 51.4	395.6 ± 49.2	378.6 ± 70.8	379.5 ± 50.0	354.9 ± 41.9*	365.8 ± 42.5	377.6 ± 43.0
OI	474.6 ± 71.8				316.2 ± 94.6*	360.6 ± 80.1*	431.1 ± 130.7
PH	7.38 ± 0.04				7.26 ± 0.06*	7.30 ± 0.04*	7.34 ± 0.03*
PaCO_2_	41.5 ± 4.0				59.2 ± 8.3*	52.5 ± 4.5*	46.9 ± 5.1*
Lac	1.25 ± 0.34				1.26 ± 0.34	1.27 ± 0.35	1.29 ± 0.35

*HR* heart rate; *MAP* mean arterial pressure; *CVP* central venous pressure; *PeakAP* peak airway pressure; *ETCO*_*2*_ end-expiratory carbon dioxide partial pressure; *TV* tidal volume; *OI* oxygenation index; *PaCO*_*2*_ carbon dioxide partial pressure; *Lac* lactic acid level

*Compared with 5 min before inflation (*P* < 0.05)

**Fig. 2:**
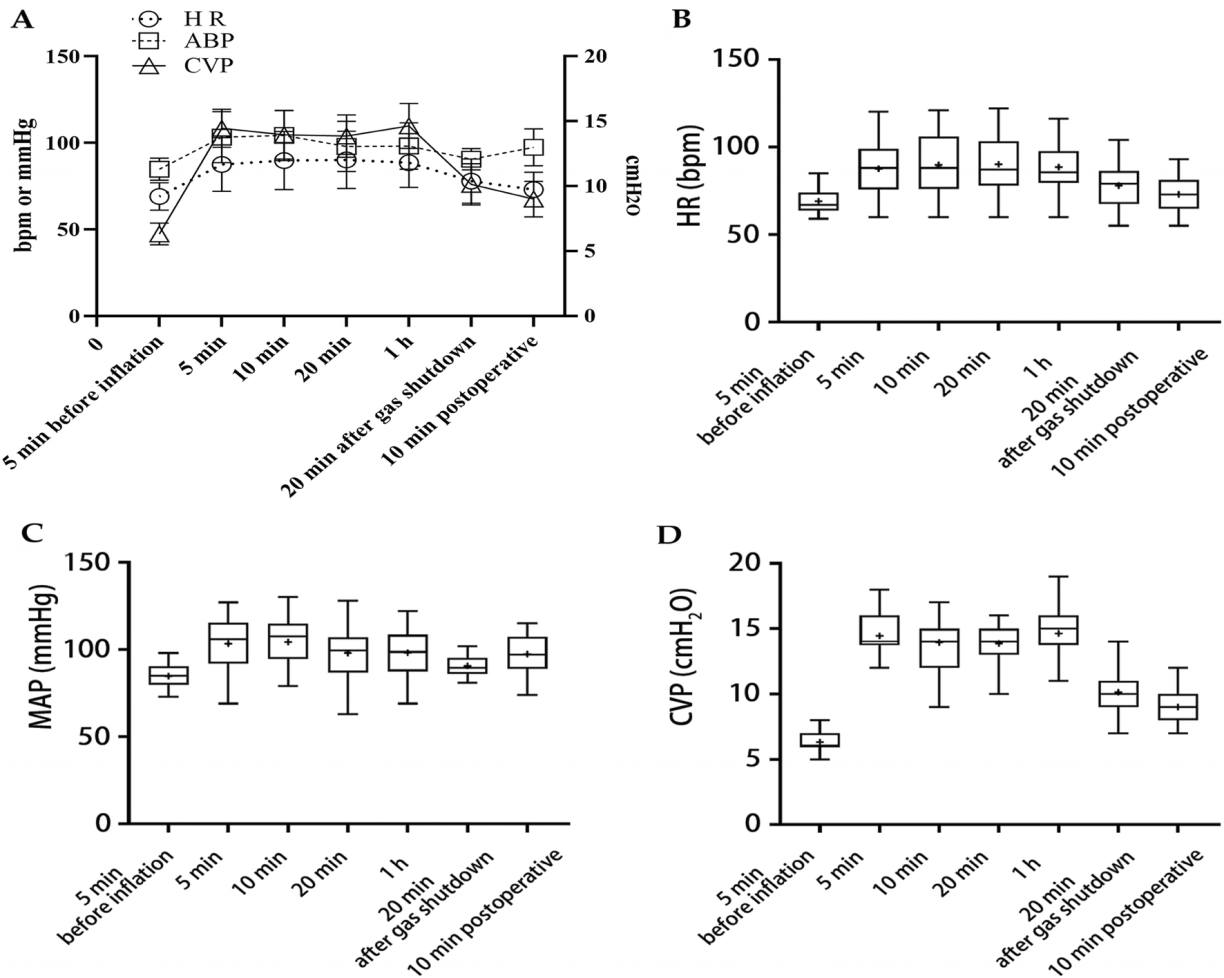
Changes in circulatory parameters after intraoperative inflation. 5 min, 10 min, 20 min, 1 h are the post-inflatable time period. **A** Showed the variations in hemodynamics after inflation. **B**–**D** Showed the intraoperative changes of HR, MAP and CVP, respectively. HR, MAP and CVP increased significantly after aeration, and decreased steadily after gas shutdown

The variations in hemodynamics after inflation are shown in Fig. 2A.The changes in HR, MAP, and CVP are shown in Fig. 2B–D respectively. HR, MAP and CVP increased immediately after inflation and exhibited no significant change during inflation; however, these values also decreased significantly after gas shutdown, although they did not return to the baseline value. When the mediastinal pressure was 10 mmHg and the pneumoperitoneum pressure was 12 mmHg, the average increase in HR was 16.5 bpm, the average increase in MAP was 18.6 mmHg, and the average increase in CVP was 8.2 cmH2O.

**Fig. 3:**
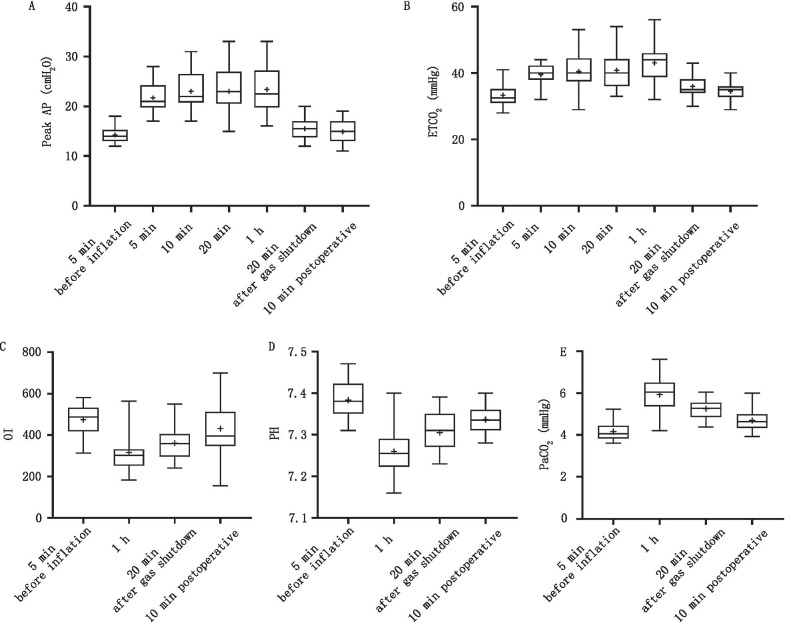
Changes of respiratory parameters after inflation during operation. 5 min, 10 min, 20 min, 1 h are the post-inflatable time period. **A**–**E** showed the intraoperative changes of Peak AP, ETCO_2_, OI, PH and PaCO_2_, respectively. After inflation, Peak AP, ETCO_2_ and PaCO_2_ increased significantly, while OI and PH decreased significantly. The baseline level can be gradually restored after gas shutdown

The changes in Peak AP, ETCO_2_, OI, PH, and PaCO_2_ are shown in Fig. 3A–E respectively. Peak AP increased immediately after inflation (Fig. 3A). The average value of Peak AP was 22.8 cmH_2_O during inflation, with a mean increase of 7.4 cmH_2_O (*P* < 0.05) 5 min after inflation. The average ETCO_2_ of 6.2 cmH_2_O (*P* < 0.05) increased 5 min after inflation (Fig. 3B), and the average value of ETCO_2_ was 41.0 mmHg during inflation. Compared with the baseline value, the TV decreased significantly at 1 h after inflation (*P* < 0.05). The OI decreased sharply at 1 h after inflation, with an average decrease of 158.6, gradually returning to close to the baseline value after gas shutdown (Fig. 3C). PaCO_2_ increased 17.7 mmHg on average at 1 h after inflation (Fig. 3E). However, there was no significant change in the blood-gas lactic acid level before and after inflation (P = NS).

The incidence of postoperative complications is shown in Table 3. 90-day mortality was 0. Anastomotic fistula occurred in 9 cases (8.6%), which were cured after vacuum sealing drainage (VSD) or routine drainage and adequate nutrition treatment. Among them, 9 cases (8.6%) required management of pleural effusion, and all of these cases were treated by indwelling thoracic tube drainage. Postoperative chylous leakage occurred in 1 case (0.9%). Approximately 300–400 ml of a chylous fluid was drained. Enteral nutrition was stopped and then restored following parenteral nutrition and adequate drainage. Postoperative atelectasis occurred in 0 cases. Postoperative Pneumonia occurred in 8 cases (7.6%), which could be cured by anti-infective treatment. Arrhythmia occurred in 4 cases (3.8%). Hoarseness was caused by recurrent laryngeal nerve injury in 19 cases (18.2%); however, such injury of the recurrent laryngeal nerve was temporary and reversible. Voice training and an intravenous drip of ganglioside 40 mg/d were used for 3 days, and the patients gradually recovered within 1 to 2 months. Only 5 patients still had hoarseness 3 months after the operation. The visual analog scale (VAS) score of all patients in the first 3 days after operation was 4.09 ± 0.57.

**Table 3:** Perioperative observation index

Parameters	Quantity	(%)
Blood loss (ml)	113.4 ± 89.9	–
operative time(min)	186.7 ± 43.9	–
Conversion to thoracotomy	1	0.9
Postoperative hospital stay	18.6 ± 8.0	–
R0 resection	–	100
The 90-day mortality	0	0
Lymph node dissection	22.5 ± 4.5	–
Complication	–	–
Anastomotic fistula	9	8.6
Pleural effusion that needs to be treated	9	8.6
Chylothorax	1	0.9
Atelectasis	0	0
Pneumonia	8	7.6
Arrhythmia	4	3.8
hoarseness	19	18.2
Hoarseness at 3 months postoperatively	5	4.8

## Discussion

Transthoracic esophagectomy has become the standard procedure for EC because it enables extensive mediastinal lymphadenectomy [19–21]. However, postoperative pulmonary complications are a major problem in transthoracic surgery and the main cause of morbidity and mortality after thoracic surgery [22]. Non-transthoracic surgery, such as THE, is another option for MIE. Single-port mediastinoscopy-assisted transesophageal hiatal esophagectomy, which was developed by Fujiwara et al., has the advantages of less potential blood loss, a shorter operative time, and fewer cardiopulmonary complications [23]. Our team improved the procedure, which was administered to two groups at the same time, and the operative time was obviously shortened. Ye et al. conducted a retrospective comparative study and suggested that left recurrent laryngeal lymph node dissection is still a major technical challenge in MIE [21]. As we previously reported [15], we entered the mediastinum through the cervical incision, inflated it to form a mediastinal cavity, and easily cleared the lymph nodes along the left recurrent laryngeal nerve while also completely clearing the subcarinal lymph nodes. A retrospective study reported that the incidence of pneumonia and arrhythmia after transthoracic minimally invasive esophagectomy was 12.4% and 14%, respectively [24]. In this study, we had a low incidence of postoperative pneumonia and arrhythmias.

**Fig. 4:**
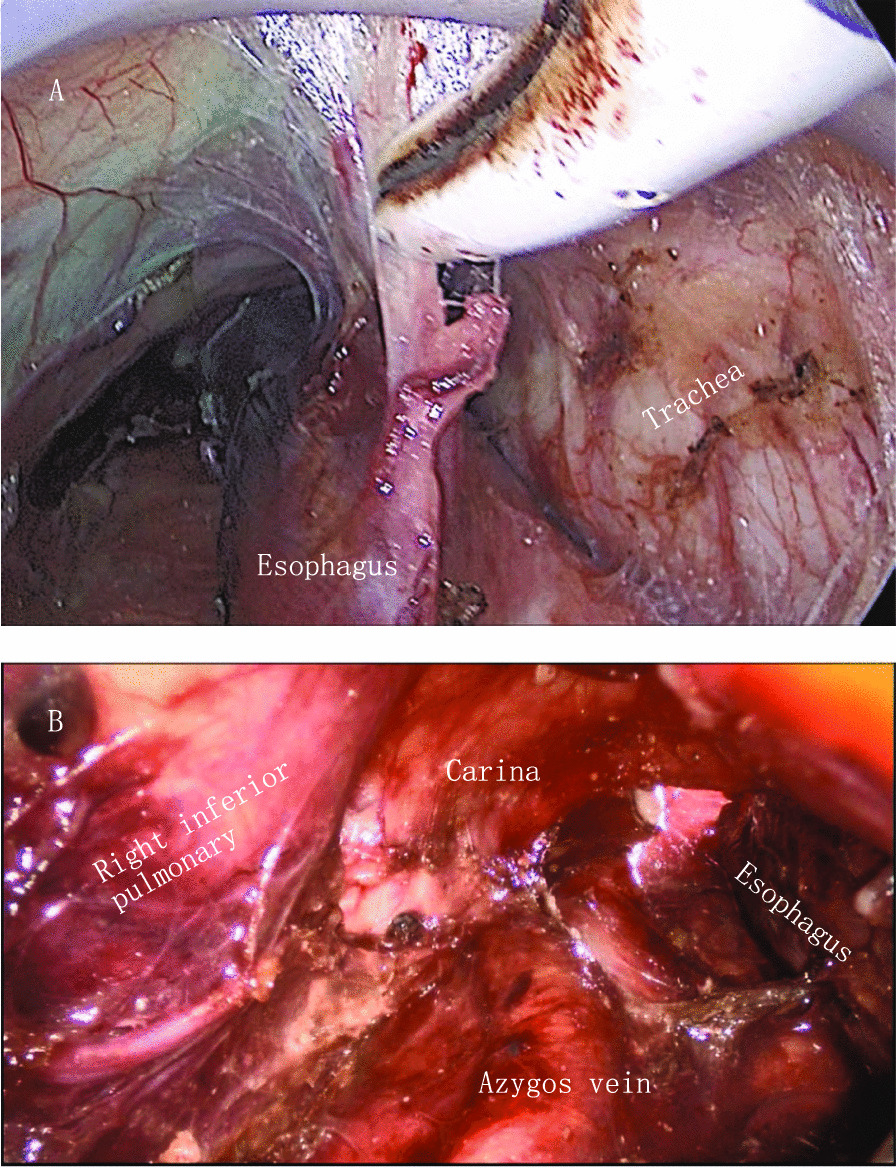
Intraoperative visual field after inflation. **A** Transcervical view in the upper mediastinum in the artificial emphysema. **B** Transcervical view when the upper and lower mediastinum penetrates

In this study, we inflated the mediastinum and abdomen at the same time to provide a clear surgical field of vision (Fig. 4). Our average operative time was 240.7 min, and the average intraoperative blood loss was 113.4 ml. Compared with thoracoabdominal endoscopy or traditional transthoracic open esophagectomy, our procedure reduced the operative and blood loss [25]. A retrospective study comparing one-lung ventilation and two-lung ventilation during MIE showed that the incidence of hypoxemia in the two-lung ventilation group was significantly lower than that in the one-lung ventilation group [26]. In our study, single-lumen endotracheal intubation was also used, but CO_2_ gas was filled into the mediastinum and abdomen simultaneously. Pneumoperitoneum and mediastinal emphysema, intraabdominal pressure, and CO_2_ absorption into the blood may cause a series of pathophysiological changes, affecting the respiratory and circulatory function of the patients and leading to postoperative cardiopulmonary complications. CO_2_ can increase the intra-abdominal and mediastinal pressures, elevate the diaphragm and compress the parietal pleura, thereby reducing lung compliance and increasing airway pressure, thus changing the pulmonary ventilation function and affecting intraoperative respiratory function [27, 28]. Hypercapnia occurs after CO_2_ is absorbed into the blood and can induce a series of stress responses (such as stimulation of the sympathetic nervous system and the release of catecholamines), stimulate the cardiovascular system, and cause an increase in arterial pressure [29]. Secondly, increased intra-abdominal pressure and mediastinal pressure can compress large blood vessels, reduce venous reflux, increase systemic vascular resistance and increase CVP. At the same time, cardiac output is reduced, and the reflex causes sympathetic nerve excitation, which eventually increases arterial pressure and then affects circulatory function during the operation [29].

This study was a retrospective study of 104 patients who successfully completed SPIMSLE. During the operation, CO_2_ was infused into the mediastinum with a mediastinal pressure of 10 mmHg and an intraperitoneal pressure of 12 mmHg. The HR, MAP and CVP increased significantly after inflation, but the values did not fluctuate and were within a safe and acceptable range during the inflation stage and gradually returned to the pre-inflation state after gas closure. No hypoxemia or respiratory inhibition was observed throughout the entire. After CO_2_ inflation, pH decreased, while PaCO_2_, peak AP and ETCO_2_ increased significantly after inflation, but these changes did not last long and returned to the normal level after the removal of CO_2_. Although PaCO_2_ increased significantly during the operation, the vital signs of most patients were stable, and there was no significant change in blood lactic acid levels, except for 2 patients who required adjustment in respiratory parameters to correct the elevated PaCO_2_. Currently, there is no clear definition of the acceptable elevation of PaCO_2_ in the clinic, so it is necessary to make a comprehensive judgment that also considers the vital signs of the patients. Although the OI decreased after inflation, it was still within a safe and acceptable range. With the deepening of the upper mediastinum and the lower mediastinum during the operation, the space of the operation was narrow, so it was necessary to control the effect of bilateral lung respiration on the mediastinal operation; moreover, the TV was artificially adjusted, so there was a significant difference between the TV at one hour after inflation and the baseline TV value. When dissociating the middle and lower esophagus during the operation, the lower mediastinal retractor should be used to lift the heart. In the study, 5 patients had hypotension or frequent premature ventricular contractions, which led to suspension of the operation. Notably, after waiting for a few minutes, the patients could continue the operation after their vital signs were stable. Therefore, when the mediastinal pressure and pneumoperitoneum pressure are 10 mmHg and 12 mmHg, respectively, this can provide a clear operative field and sufficient operating space, and the operation is safe under standard operation conditions.

Minimally invasive surgery should be the first choice for patients with EC. Compared with MIE via a transthoracic approach, THE is considered to be less invasive and more tolerable, especially in patients with severe complications [9, 30]. However, when the patient has comorbid diseases, such as severe chest adhesions, chest deformities, poor cardiopulmonary function, poor tolerance of one-lung ventilation, or a history of major right lung surgery [15, 31], transthoracic MIE is very difficult for surgeons and patients, and SPIMSLE may be a better choice.

In this study, we successfully completed the operation in 104 patients, there was no perioperative death, and the postoperative pathology confirmed that the R0 resection rate was 100%. The incidence of postoperative anastomotic fistula was similar to that of MIE via the transthoracic approach (5.0 ~ 12%) [4, 32]. Compared with minimally invasive transthoracic esophagectomy [4], the incidence of postoperative hoarseness is still higher and needs to be improved There are still some limitations of this study. First, this study was retrospective, and we did not include patients with other EC operations as a control group. In subsequent research, a well-designed randomized controlled trial should be conducted to comprehensively evaluate the efficacy and safety of this new surgical method. Second, we did not evaluate the long-term quality of life of the patients after surgery. All these limitations should be addressed in future studies.

## Conclusion

When the inflation pressures in the mediastinum and abdomen were 10 mmHg and 12 mmHg, respectively, we safely completed surgery without respiratory and circulatory inhibition. In summary, our results show that our single-port inflatable mediastinoscopy with simultaneous laparoscopic radical resection of EC is technically safe and does not increase perioperative complications.

## Data Availability

The datasets used and/or analysed during the current study are available from the corresponding author on reasonable request.

## Abbreviations

EC: Esophageal cancer
MIE: Minimally invasive esophagectomy
THE: Transhiatal esophagectomy
SPIMSLE: Single-port inflatable mediastinoscopy simultaneous laparoscopic radical esophagectomy
CVP: Central venous pressure
CO_2_: Carbon dioxide
HR: Heart rate
MAP: Mean arterial pressure
peak AP: Peak airway pressure
ETCO_2_: End-expiratory CO_2_ partial pressure
FiO_2_: Inhaled oxygen concentration
TV: Tidal volume
OI: Oxygenation index
VSD: Vacuum sealing drainage
VAS: Visual analog scale
